# Ultrasonographic Microcalcifications in Metastatic Lymph Nodes of Papillary Thyroid Carcinoma: A Sonopathological Study

**DOI:** 10.3390/diagnostics16071048

**Published:** 2026-03-31

**Authors:** Adile Begüm Bahçecioğlu, Meliha Merve Ekelik Özyılmaz, Muhammed Şamil Özlü, Serpil Dizbay Sak, Sevim Güllü, Murat Faik Erdoğan

**Affiliations:** 1 Department of Endocrinology and Metabolism, Gülhane Training and Research Hospital, Ankara 06010, Türkiye; 2 Department of Internal Medicine, Sincan Training and Research Hospital, Ankara 06930, Türkiye; mervekelik@gmail.com; 3 Department of Pathology, School of Medicine, Ankara University, Ankara 06100, Türkiye; msozlu@ankara.edu.tr (M.Ş.Ö.); serpil.d.sak@medicine.ankara.edu.tr (S.D.S.); 4 Department of Endocrinology and Metabolism, School of Medicine, Ankara University, Ankara 06100, Türkiye; sevim.gullu@temd.org.tr (S.G.); murat.erdogan@temd.org.tr (M.F.E.)

**Keywords:** thyroid neoplasms, papillary thyroid carcinoma, lymphatic metastasis, lymph nodes, ultrasonography, pathology, diagnostic imaging

## Abstract

**Background/Objectives**: Ultrasonographic microcalcifications are highly specific imaging features of malignancy in both thyroid nodules and metastatic cervical lymph nodes in papillary thyroid carcinoma (PTC). However, although their histopathological correlates in thyroid nodules have been extensively investigated, the pathological substrates underlying microcalcifications in metastatic lymph nodes remain poorly defined, particularly for ultrasound-based diagnostic interpretation. **Methods**: This retrospective sonopathological cohort study included 32 patients with PTC, contributing 70 metastatic cervical lymph nodes. Lymph nodes were classified based on the presence or absence of microcalcifications detected by ultrasonography. Histopathological features—psammoma bodies, hyalinization, stromal calcification, cystic degeneration, and papillary formation—were systematically re-evaluated by pathologists blinded to ultrasonographic findings using a predefined semi-quantitative grading protocol. Microcalcification-positive metastatic lymph nodes were additionally compared with a reference cohort of microcalcification-positive thyroid nodules (*n* = 30). **Results**: Ultrasonographic microcalcifications were identified in 30 of 70 metastatic lymph nodes (42.9%). Microcalcification-positive lymph nodes demonstrated significantly higher frequencies of psammoma bodies (56.7% vs. 17.5%, *p* = 0.001), moderate-to-extensive psammoma bodies (40.0% vs. 12.5%, *p* = 0.003), stromal calcification (30.0% vs. 0.0%, *p* < 0.001), and cystic degeneration (80.0% vs. 12.5%, *p* < 0.001) compared with microcalcification-negative nodes. Notably, 43.3% of microcalcification-positive nodes lacked moderate-to-extensive psammoma bodies, indicating heterogeneous pathological correlates. Comparative analysis revealed no significant differences between microcalcification-positive nodes and thyroid nodules in the prevalence or extent of psammoma bodies or stromal calcification; however, hyalinization was significantly more frequent and extensive in thyroid nodules (both *p* < 0.001). **Conclusions**: Ultrasonographic microcalcifications in metastatic lymph nodes of PTC reflect heterogeneous histopathological correlates beyond psammoma bodies alone. These findings highlight the importance of anatomically informed, context-specific interpretation of microcalcifications in ultrasound-based diagnostic practice.

## 1. Introduction

Papillary thyroid carcinoma (PTC) is the most common endocrine malignancy and accounts for the majority of thyroid cancers. Its incidence has markedly increased over recent decades, with an estimated 44,020 new thyroid cancer cases reported in the United States in 2024 [1]. It is characterized by a high propensity for cervical lymph node metastasis, which plays a critical role in disease staging, risk stratification, and surgical planning [1,2]. High-resolution ultrasonography (US) is the primary imaging modality for the preoperative evaluation of thyroid nodules and cervical lymph nodes, owing to its wide availability, cost-effectiveness, and high diagnostic performance [1].

Metastatic cervical lymph nodes play a critical role in the staging and management of thyroid carcinoma, and ultrasonography remains the primary imaging modality for their evaluation [1]. Among ultrasonographic features associated with malignancy, microcalcifications are considered among the most specific findings in thyroid nodules, with reported sensitivities ranging from 84% to 97% [3,4,5]. In the context of cervical lymph nodes, the presence of microcalcifications is likewise regarded as a strong indicator of metastatic involvement [1,6,7]. Several studies have demonstrated that microcalcifications maintain a high diagnostic value even after adjustment for other sonographic parameters, including lymph node size, shape, echogenicity, and loss of the fatty hilum. In particular, Rosario et al. showed that microcalcifications are among the most specific ultrasonographic features for metastatic lymph nodes in patients with PTC, independently differentiating malignant from benign nodal disease [8]. Supporting these findings, a large retrospective study by Liu et al. involving patients with papillary thyroid microcarcinoma demonstrated that calcification, along with cystic change, round shape, loss of echogenic fatty hilum, and abnormal vascularity, remained an independent predictor of lymph node metastasis in multivariate analysis [6]. Nevertheless, despite the recognized specificity of microcalcifications and other sonographic criteria, differentiating metastatic from benign cervical lymph nodes may remain challenging due to overlapping imaging features and variable expression. Collectively, these data underscore the central role of microcalcifications in the ultrasonographic assessment of cervical lymph nodes, leading to their widespread incorporation into clinical decision-making algorithms that guide fine-needle aspiration and surgical management in patients with PTC.

Histopathologically, ultrasonographic microcalcifications in thyroid nodules have traditionally been attributed to psammoma bodies—concentrically laminated calcified structures characteristic of PTC [9]. However, emerging evidence suggests that echogenic microfoci in thyroid nodules may not be exclusively attributable to psammoma bodies and may also reflect stromal calcification, fibrosis, or degenerative changes [10,11]. These observations raise important questions regarding the biological and histopathological heterogeneity underlying ultrasonographic microcalcifications.

In contrast to thyroid nodules, the histopathological correlates of ultrasonographic microcalcifications in metastatic lymph nodes remain poorly defined. Despite their established diagnostic significance, it is unclear whether microcalcifications observed in metastatic lymph nodes represent the same pathological substrates as those seen in primary thyroid tumors, or whether they arise from distinct stromal or degenerative processes within the nodal microenvironment. Importantly, direct sonopathological correlation studies focusing specifically on metastatic lymph nodes are scarce. Limited histopathological investigations have suggested that microcalcifications within metastatic lymph nodes may correspond to psammoma bodies, intralymphatic tumor deposits with dystrophic calcification, or stromal calcifications associated with necrosis or fibrosis; however, these observations are based on small series and lack systematic sonopathological correlation [12,13].

From a conceptual standpoint, the term “microcalcification” is a sonographic descriptor without a direct, specific histopathological correlate. Although traditionally interpreted as a surrogate marker for psammoma bodies in thyroid nodules, accumulating evidence suggests that punctate echogenic foci detected on ultrasonography may reflect a heterogeneous spectrum of underlying pathological substrates. In clinical practice, the interpretation of microcalcifications in cervical lymph nodes is largely extrapolated from criteria established for thyroid nodules, despite fundamental anatomical and microenvironmental differences between the two sites. This conceptual generalization raises the possibility that identical sonographic appearances may correspond to distinct histopathological processes depending on tissue context. Therefore, the aim of the present study was to investigate the histopathological correlates of ultrasonographically detected microcalcifications in metastatic lymph nodes of patients with PTC. In addition, we sought to compare these findings with the histopathological features of microcalcification-positive thyroid nodules to determine whether the sonopathological basis of microcalcifications differs between primary tumors and metastatic lymph nodes.

## 2. Materials and Methods

### 2.1. Study Design and Population

This retrospective sonopathological cohort study was conducted at Ankara University Faculty of Medicine, Departments of Endocrinology and Metabolism and Pathology. Clinical and imaging data were collected from patients who underwent surgery between 2017 and 2019 for PTC with cervical lymph node metastasis. Pathological reassessment for the present study was completed in 2024. Patients and metastatic lymph nodes were identified retrospectively from surgical and pathology records. Patients with PTC who underwent cervical lymph node dissection due to metastatic lymph node involvement were eligible for inclusion. Metastatic lymph nodes and their corresponding thyroid nodules, obtained from surgical specimens, were evaluated.

Patients were included if:

(1) Preoperative cervical ultrasonography demonstrated evaluable cervical lymph nodes;
(2) Lymph node dissection confirmed metastatic PTC on histopathological examination;
(3) Adequate histological material was available for detailed pathological reassessment.

Lymph nodes without sufficient sonographic–pathologic correlation were excluded from the analysis.

The unit of analysis in this study was the individual lymph node. As multiple metastatic lymph nodes were obtained from some patients, more than one observation could originate from the same individual. Therefore, the analysis was performed at the lymph node level rather than at the patient level.

A subset of microcalcification-positive thyroid nodules included in the present study was derived from a previously published cohort [11]. In the current analysis, these thyroid nodules were used exclusively as a reference group for comparison, and no primary outcome analyses from the prior publication were repeated. The primary focus of the present study was the histopathological correlates of ultrasonographic microcalcifications in metastatic lymph nodes.

### 2.2. Ultrasonographic Evaluation and Grouping

Preoperative cervical ultrasonography examinations were reviewed retrospectively. Ultrasonographic examinations were performed by experienced operators using high-frequency linear transducers, in accordance with institutional protocols. Ultrasonographic images were independently reviewed by MFE and ABB, both blinded to the histopathological findings at the time of image evaluation. Metastatic lymph nodes were classified according to the presence or absence of ultrasonographic microcalcifications.

Two predefined comparisons were performed:

Microcalcification-positive versus microcalcification-negative metastatic lymph nodes;

Microcalcification-positive metastatic lymph nodes versus microcalcification-positive thyroid nodules (comparative reference analysis).

**Figure 1:**
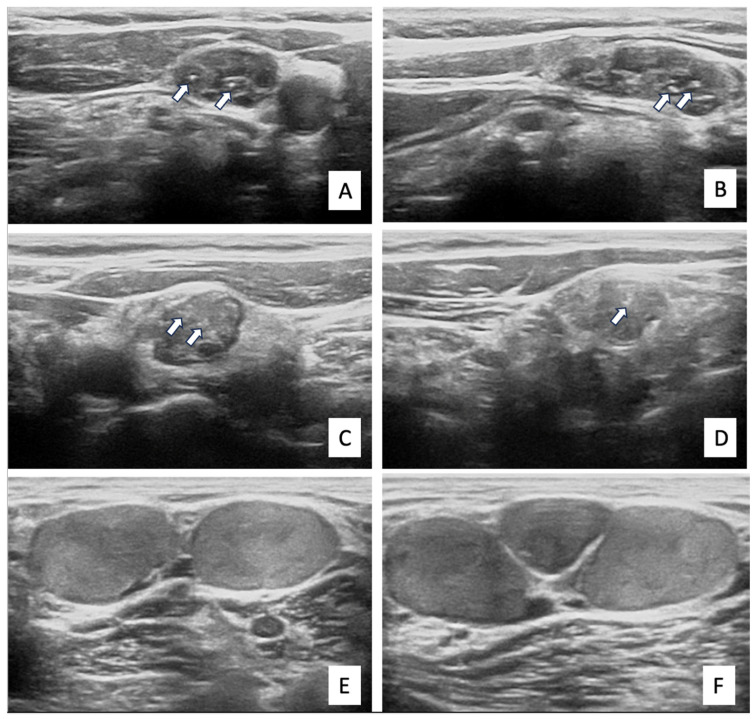
Representative ultrasonographic images of metastatic cervical lymph nodes with and without microcalcifications. (**A**,**B**) Right level III lymph node with microcalcifications on (**A**) axial and (**B**) longitudinal sections. (**C**,**D**) Left level III lymph node with microcalcifications on (**C**) axial and (**D**) longitudinal sections. (**E**,**F**) Right level IV lymph node without microcalcifications on (**E**) axial and (**F**) longitudinal sections. **Arrows indicate ultrasonographic microcalcifications**.

Ultrasonographic microcalcifications were defined as punctate echogenic foci without posterior acoustic shadowing, in accordance with established ultrasound criteria. Representative ultrasonographic images of metastatic lymph nodes with and without microcalcifications are provided in Figure 1.

### 2.3. Histopathological Evaluation

All histopathological slides obtained from cervical lymph node dissections and thyroidectomy specimens were retrospectively re-evaluated by two pathologists (S.D.S., M.Ş.Ö.) who were blinded to the ultrasonographic findings. Reassessment was performed according to a predefined study-specific protocol designed to ensure uniform evaluation of histopathological features relevant to ultrasonographic microcalcifications.

Metastatic lymph nodes and thyroid nodules were examined on hematoxylin and eosin-stained sections. The presence and extent of the following histopathological features were systematically assessed: psammoma bodies, hyalinization, stromal calcification, cystic degeneration, and papillary formation.

Each feature was graded semi-quantitatively as absent, mild, moderate, or extensive, based on its relative proportion within the examined tissue section. A semi-quantitative grading approach was adopted to capture not only the presence but also the relative extent of histopathological features, as binary classification may inadequately reflect the spectrum of pathological changes underlying ultrasonographic findings. For analytical purposes, moderate-to-extensive involvement was also evaluated to identify features more likely to contribute to detectable echogenic foci on ultrasonography and to enhance discrimination between comparison groups.

Psammoma bodies were identified by their characteristic lamellated shape, size and localization within papillary cores. Stromal calcifications were distinguished by their irregular, non-laminated appearance within fibrotic or hyalinized stroma. Both features were graded as extensive when easily recognized at low magnification, absent when not identified, and mild when detectable only after careful search. Hyalinization was defined as hypocellular eosinophilic stroma between tumor cell groups and graded similarly: extensive when abundant in most low-power fields, absent when only delicate fibrovascular stroma was observed, and mild when identified only on close examination. Cystic degeneration was defined as cavitary spaces within tumor deposits, either empty or containing papillary structures, macrophages, or colloid. The presence of papillae was defined as papillary structures with delicate fibrovascular cores lined by typical tumor cells. These features were graded using the same criteria; findings intermediate between extensive and mild were classified as moderate for all parameters.

**Figure 2:**
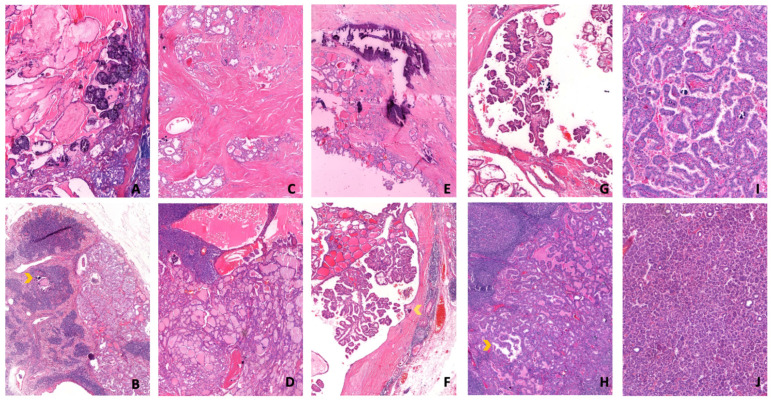
Representative histopathological features of metastatic cervical lymph nodes illustrating the semi-quantitative grading system. (**A**–**J**) Histopathological features of PTC metastases (All figures (**H**,**E**,**A**,**B**) 10×, (**C**–**J**) 5×). Psammoma bodies: (**A**): extensive, (**B**): mild (arrowhead); Hyalinization: (**C**): extensive, (**D**): mild; Stromal calcification: (**E**): extensive, (**F**): mild (arrowhead); Cystic degeneration: (**G**): extensive, (**H**): mild (arrowhead); Papillary formation: extensive, (**I**): mild.

Representative examples of the evaluated histopathological features and the semi-quantitative grading system are illustrated in Figure 2.

### 2.4. Statistical Analysis

Categorical variables were expressed as frequencies and percentages. Comparisons between microcalcification-positive and microcalcification-negative metastatic lymph nodes were performed using appropriate statistical tests. A two-sided *p*-value of <0.05 was considered statistically significant.

## 3. Result

### 3.1. Patient and Lymph Node Characteristics

A total of 32 patients with PTC, contributing 70 metastatic lymph nodes, were included in the study. Among the metastatic lymph nodes, 30 (42.9%) were microcalcification-positive and 40 (57.1%) were microcalcification-negative on ultrasonographic evaluation. The median age of the patients was 46 years (range, 24–77 years), and 75% were female (*n* = 24). The mean maximal diameter of metastatic lymph nodes was 14.2 ± 7.3 mm. Regarding histological subtypes of PTC, 22 patients had the classic subtype, six patients had the classic subtype with infiltrative follicular features, and three patients had the diffuse sclerosing subtype.

### 3.2. Histopathological Features of Metastatic Lymph Nodes According to Microcalcification Status

The histopathological features of metastatic lymph nodes, stratified by ultrasonographic microcalcifications, are summarized in Table 1.

As shown in Table 1, metastatic lymph nodes with ultrasonographic microcalcifications exhibited distinct histopathological characteristics compared with those without microcalcifications. The presence and extent of psammoma bodies, as well as the occurrence of stromal calcification and cystic degeneration, were significantly more common in microcalcification-positive lymph nodes, whereas papillary formation did not differ significantly between the two groups. Notably, a substantial proportion of microcalcification-positive metastatic lymph nodes lacked moderate-to-extensive psammoma bodies, suggesting that microcalcifications detected on ultrasound frequently occurred in the absence of prominent classical calcific structures. In these nodes, stromal calcification and cystic degeneration were often observed concurrently, suggesting that combined degenerative and stromal processes may contribute to the sonographic appearance of punctate echogenic foci.

### 3.3. Histopathological Comparison of Microcalcification-Positive Thyroid Nodules and Metastatic Lymph Nodes

The comparative histopathological features of microcalcification-positive thyroid nodules and microcalcification-positive metastatic lymph nodes are summarized in Table 2.

While the overall prevalence of psammoma bodies, stromal calcification, cystic degeneration, and papillary formation did not differ significantly between microcalcification-positive metastatic lymph nodes and thyroid nodules, hyalinization was significantly more frequent and more extensive in microcalcification-positive thyroid nodules (Table 2).

## 4. Discussion

The present study provides a comprehensive sonopathological evaluation of ultrasonographic microcalcifications in metastatic lymph nodes of PTC and demonstrates that their histopathological correlates extend beyond psammoma bodies alone. Although microcalcification-positive metastatic lymph nodes were more frequently associated with psammoma bodies than microcalcification-negative nodes, stromal calcification and cystic degeneration emerged as prominent distinguishing features. Moreover, comparison with microcalcification-positive thyroid nodules revealed distinct histopathological patterns, particularly regarding the presence and extent of hyalinization. These findings indicate that, despite a shared ultrasonographic appearance, microcalcifications in metastatic lymph nodes and primary thyroid tumors reflect different underlying pathological features, underscoring the importance of site-specific sonohistopathological interpretation.

In the intranodal comparison, microcalcification-positive metastatic lymph nodes showed significantly higher rates of stromal calcification and cystic degeneration than microcalcification-negative nodes. This finding indicates that ultrasonographic microcalcifications in metastatic lymph nodes likely reflect a heterogeneous range of tumor-associated pathological processes within the nodal microenvironment, rather than representing exclusively true psammoma bodies. As metastatic deposits enlarge, progressive tumor necrosis, cystic degeneration, stromal remodeling, and secondary calcification may collectively contribute to the formation of echogenic foci detectable on ultrasonography. Accordingly, microcalcifications observed in metastatic lymph nodes are better interpreted as a composite of tumor-related degenerative changes and stromal responses rather than a single uniform histological substrate. The lymph node constitutes a distinct microenvironment characterized by specialized stromal architecture, immune cell populations, and lymphatic flow dynamics, all of which differ substantially from those of the thyroid parenchyma [14]. These factors may influence tumor growth patterns, stromal remodeling, and secondary degenerative changes within metastatic deposits. Consequently, calcific and cystic changes arising in metastatic lymph nodes may reflect site-specific interactions between tumor cells and the nodal microenvironment rather than direct analogues of calcific processes observed in primary thyroid tumors.

Early sonopathological correlation studies emphasized a direct correspondence between punctate echogenic foci and psammoma bodies in metastatic cervical lymph nodes [12]. However, these studies were limited by small sample sizes, qualitative histological assessment, and lower-resolution ultrasound technology. More recent investigations have highlighted the morphological complexity of metastatic lymph nodes, including frequent cystic degeneration and stromal remodeling, supporting a broader pathological basis for ultrasonographic echogenic foci [13]. Our findings extend this evolving concept by providing quantitative evidence that stromal calcification and cystic degeneration are significantly enriched in microcalcification-positive metastatic lymph nodes, highlighting potential ultrasound–histology discordance when microcalcifications are interpreted as a uniform marker of psammoma bodies alone.

The stromal and degenerative changes observed in microcalcification-positive metastatic lymph nodes may also reflect the biological behavior of the primary tumor and its mode of spread. Jung et al. demonstrated that specific invasive growth patterns in PTC, including isolated glands and lateral tubular growth, are strong independent predictors of lymph node metastasis, underscoring the role of tumor–stroma interactions and lymphatic dissemination in nodal involvement [15]. Similarly, Chung et al. reported that aggressive histomorphological features such as hobnail growth, loss of cellular polarity, micropapillary architecture, infiltrative tumor borders, and prominent stromal fibrosis are significantly associated with nodal metastasis [16]. In this context, the enrichment of stromal calcification, hyalinization, and cystic degeneration identified in our study may represent downstream histopathological consequences of aggressive and infiltrative tumor growth within the lymph node microenvironment rather than direct analogues of calcific processes seen in primary thyroid tumors.

A key and novel contribution of the present study is the direct quantitative comparison of microcalcification-positive metastatic lymph nodes and microcalcification-positive thyroid nodules within a unified sonopathological framework. To our knowledge, this is the first study to demonstrate that, despite similar ultrasonographic appearances, the histopathological correlates underlying microcalcifications differ substantially between these two anatomical compartments. This divergence suggests that echogenic foci grouped under the shared term “microcalcifications” may be sonographically similar yet histopathologically non-interchangeable across sites. In metastatic lymph nodes, these foci may be relatively coarser, more heterogeneous, and arise predominantly from secondary stromal and degenerative processes, whereas in thyroid nodules they more often reflect tumor-intrinsic calcific features. This observation highlights a potential limitation of current ultrasonographic lexicon and diagnostic algorithms, which often extrapolate interpretations from thyroid nodules to lymph nodes without accounting for anatomical context.

Several limitations should be acknowledged. The retrospective design may have introduced selection bias and limited the uniformity of available data. In addition, microcalcification-positive lymph nodes and thyroid nodules were not derived from the same patients, precluding direct paired comparisons and necessitating group-level interpretation. Another limitation of the study is that the analysis was conducted at the lymph node level, and multiple nodes were obtained from some patients. This may introduce potential intra-patient correlation, which could affect the assumption of complete statistical independence between observations. The semi-quantitative nature of histopathological scoring, while performed using predefined criteria by experienced endocrine pathologists, may also introduce observer variability. Additionally, survival and long-term outcome data were not assessed, as the primary aim of the study was to investigate sonopathological correlations rather than prognostic impact. Additionally, survival and long-term outcome data were not assessed, as the primary aim of the study was to investigate sonopathological correlations rather than prognostic impact. Finally, the relatively small sample size, particularly in subgroup analyses, may have reduced statistical power and limited the ability to detect more subtle differences. Despite these limitations, the systematic and quantitative approach of the present study provides robust evidence supporting a context-dependent interpretation of ultrasonographic microcalcifications. Additionally, inherent scale mismatch between ultrasonographic imaging and histopathological sectioning should be considered, as punctate echogenic foci detected in vivo may represent composite or spatially heterogeneous microscopic structures that are not fully captured within individual histological sections.

## 5. Conclusions

In conclusion, ultrasonographic microcalcifications in metastatic lymph nodes of PTC are associated with heterogeneous histopathological substrates extending beyond psammoma bodies alone. The enrichment of stromal calcification, cystic degeneration, and hyalinization underscores the influence of the nodal microenvironment on the formation of echogenic foci. Furthermore, the direct comparison with microcalcification-positive thyroid nodules demonstrates that identical sonographic findings may reflect distinct pathological processes across different anatomical sites. These results refine the conventional interpretation of microcalcifications and underscore the need for anatomically informed, context-specific sonographic assessment in both clinical practice and future research.

## Figures and Tables

**Table 1:** Histopathological features of metastatic lymph nodes according to the presence of ultrasonographic microcalcifications.

Histopathological Features	No Microcalcifications (*n* = 40, %)	Microcalcifications Present (*n* = 30, %)	*p* Value
Presence of psammoma bodies	7 (17.5)	17 (56.7)	0.001
Moderate-to-extensive psammoma bodies	5 (12.5)	12 (40.0)	0.003
Presence of hyalinization	9 (22.5)	13 (43.3)	0.063
Moderate-to-extensive hyalinization	0 (0.0)	2 (6.7)	0.084
Presence of stromal calcification	0 (0.0)	9 (30.0)	<0.001
Presence of cystic degeneration	5 (12.5)	24 (80.0)	<0.001
Presence of papillary formation	30 (75.0)	26 (86.7)	0.227

**Table 2:** Comparison of histopathological features between microcalcification-positive metastatic lymph nodes and thyroid nodules.

Histopathological Features	Microcalcification-Positive Lymph Nodes (*n* = 30, %)	Microcalcification-Positive Thyroid Nodules (*n* = 30, %)	*p* Value
Presence of psammoma bodies	17 (56.7)	21 (52.5)	0.729
Moderate-to-extensive psammoma bodies	12 (40.0)	13 (32.5)	0.501
Presence of hyalinization	13 (43.3)	34 (85.0)	<0.001
Moderate-to-extensive hyalinization	2 (6.7)	17 (42.5)	<0.001
Presence of stromal calcification	9 (30.0)	14 (35.0)	0.659
Presence of cystic degeneration	24 (80.0)	26 (65.0)	0.169
Presence of papillary formation	26 (86.7)	30 (75.0)	0.227

## Data Availability

The data presented in this study are available on request from the corresponding author (A.B.B.) due to privacy and ethical restrictions.
